# First-principles demonstration of band filling-induced significant improvement in thermodynamic stability and mechanical properties of Sc$_{1-x}$Ta$_{x}$B$_{2}$ solid solutions

**DOI:** 10.1038/s41598-023-37642-8

**Published:** 2023-06-28

**Authors:** Kunpot Mopoung, Annop Ektarawong, Thiti Bovornratanaraks, Björn Alling

**Affiliations:** 1 Extreme Condition Physics Research Laboratory and Center of Excellence in Physics of Energy Materials, Department of Physics, Faculty of Science, Chulalongkorn University, Bangkok, 10330 Thailand; 2 Chula Intelligent and Complex Systems, Faculty of Science, Chulalongkorn University, Bangkok, 10330 Thailand; 3 Theoretical Physics Division, Department of Physics, Chemistry and Biology (IFM), Linköping University, SE-581 83 Linköping, Sweden

**Keywords:** Mechanical properties, Metals and alloys, Atomistic models, Electronic structure

## Abstract

Mixtures of different metal diborides in the form of solid solutions are promising materials for hard-coating applications. Herein, we study the mixing thermodynamics and the mechanical properties of AlB$_{2}$-structured Sc$_{1-x}$Ta$_{x}$B$_{2}$ solid solutions using the first-principles method, based on the density functional theory, and the cluster-expansion formalism. Our thermodynamic investigation reveals that the two diborides readily mix with one another to form a continuous series of stable solid solutions in the pseudo-binary TaB$_{2}$ $-$ScB$_{2}$ system even at absolute zero. Interestingly, the elastic moduli as well as the hardness of the solid solutions show significant positive deviations from the linear Vegard’s rule evaluated between those of ScB$_{2}$ and TaB$_{2}$. In case of Sc$_{1-x}$Ta$_{x}$B$_{2}$, the degrees of deviation from such linear trends can be as large as 25, 20, and 40% for the shear modulus, the Young’s modulus, and the hardness, respectively. The improvement in the stability as well as the mechanical properties of Sc$_{1-x}$Ta$_{x}$B$_{2}$ solid solutions relative to their constituent compounds is found to be related to the effect of electronic band filling, induced upon mixing TaB$_{2}$ with ScB$_{2}$. These findings not only demonstrate the prominent role of band filling in enhancing the stability and the mechanical properties of Sc$_{1-x}$Ta$_{x}$B$_{2}$, but also it can potentially open up a possibility for designing stable/metastable metal diboride-based solid solutions with superior and widely tunable mechanical properties for hard-coating applications.

## Introduction

Double-metal diborides with chemical formula of M$_{1-x}^{\prime }$M$_{x}^{\prime \prime }$B$_{2}$, where M$^{\prime }$ and M$^{\prime \prime }$ are two distinct metallic elements (Mg, Al, Sc, Y, Ti, Zr, Hf, V, Nb, Ta, Cr, Mo, W, Ru, Os, and Re, for example) and 0 $\leqslant$ *x* $\leqslant$ 1, have increasingly gained interest in the recent years as promising hard-coating materials for cutting tools, thanks to their high thermal and chemical stabilities as well as good mechanical properties^1–13^. It has lately been shown from the theoretical and experimental aspects that, by adding the second metallic element M$^{\prime \prime }$ to the diboride compounds M$^{\prime }$B$_{2}$, the stabilities and properties of the resulting double-metal diborides M$_{1-x}^{\prime }$M$_{x}^{\prime \prime }$B$_{2}$ can be significantly improved and thus become superior to those of the constituent diboride compounds, either M$^{\prime }$B$_{2}$ or M$^{\prime \prime }$B$_{2}$^3–8,10–13^. For instance, alloying TaB$_{2}$ with ZrB$_{2}$ to form thin films of Ta$_{1-x}$Zr$_{x}$B$_{2}$ results in improvement in hardness and toughness of the films, relative to those of TaB$_{2}$ and ZrB$_{2}$ films^7,10,13^. Another example is that introducing Cr (Al) atoms into ZrB$_{2}$ (TiB$_{2}$) films increases wear, oxidation, and corrosion resistances of the films, and the mechanical properties of the resulting off-stoichiometric films of Zr$_{1-x}$Cr$_{x}$B$_{y}$ (Ti$_{1-x}$Al$_{x}$B$_{y}$) can be improved or tuned *via* controlling the films’ composition^6,8,11^. The enhancement of stabilities and mechanical properties of M$_{1-x}^{\prime }$M$_{x}^{\prime \prime }$B$_{2}$ with respect to M$^{\prime }$B$_{2}$ and M$^{\prime \prime }$B$_{2}$ has been suggested to be directly related to the changes in the number of valence electrons filling bonding and antibonding electronic states of the material^14–16^, controlled by variation of M$^{\prime }$B$_{2}$ and M$^{\prime \prime }$B$_{2}$ contents, as recently demonstrated in Sc$_{1-x}$V$_{x}$B$_{2}$ exhibiting superior thermodynamic stability and mechanical properties, especially hardness, as compared to ScB$_{2}$ and VB$_{2}$^12^.

Apart from the double-metal diborides, such a band-filling scenario has theoretically been found to play a crucial role in determining the thermodynamic stability and mechanical properties of some single-metal diborides − for example, AlB$_{2}$^17^ and TaB$_{2}$^18,19^. In the case of TaB$_{2}$, the effect of band filling can be triggered by partial substitution of vacancies for B atoms in the diboride. Despite the increase in the number of broken bonds around the vacancies tending to destabilize the diboride, the replacement of some B atoms in TaB$_{2}$ by vacancies reduces the number of electrons occupying the diboride’s antibonding states enhancing its stability. The two effects arising from the formation of vacancies on the boron sublattice of TaB$_{2}$ counterbalance and eventually result in stabilization of B-deficient TaB$_{2-x}$ over a small range of *x* (0.167 $\lesssim$ *x* $\lesssim$ 0.25) in thermodynamic equilibrium. Besides, the shear strength, stiffness, hardness of thermodynamically stable TaB$_{2-x}$ are superior to those of TaB$_{2}$^18,19^. Hypothetically, replacing some Ta atoms of TaB$_{2}$ by any group-III or group-IV metallic element (Sc, Y, Ti, Zr, or Hf) to form solid solutions of Ta-containing double-metal diboride should as well yield a decrease in the number of electron occupying the antibonding states of the material without a need for forming the vacancies, and thus give rise to the aforementioned band filling-induced enhancement of thermodynamic stability and mechanical properties of the solid solutions with respect to their constituent compounds, as theoretically predicted for Sc$_{1-x}$V$_{x}$B$_{2}$ solid solutions.

Most often, Ta-containing double-metal diborides, represented by mixtures of TaB$_{2}$ and group-IV metal diboride (TiB$_{2}$, ZrB$_{2}$, HfB$_{2}$) are of interest and considered in the literature^7,10,13,16,20–26^, whereas solid solutions of TaB$_{2}$ and group-III metal diboride (ScB$_{2}$ or YB$_{2}$) are much less studied^2,27,28^. We are therefore inspired to examine using first-principles approaches the mixing thermodynamics of ScB$_{2}$ and TaB$_{2}$, both of which crystallize in the hexagonal space group of *P*6/*mmm* (AlB$_{2}$-type structure), as well as the mechanical properties of Sc$_{1-x}$Ta$_{x}$B$_{2}$ solid solutions. Our thermodynamic considerations reveal a mixing tendency of Sc and Ta atoms, residing on the metal sublattice of Sc$_{1-x}$Ta$_{x}$B$_{2}$. This thus enables formation of single-phase solid solutions of Sc$_{1-x}$Ta$_{x}$B$_{2}$ across the entire composition range (0 $\leqslant$ *x* $\leqslant$ 1) at low temperatures. Interestingly, the values of the shear and Young’s moduli as well as the hardness of Sc$_{1-x}$Ta$_{x}$B$_{2}$ are observed to largely deviate in the positive direction from the linear mixing trends drawn between those ScB$_{2}$ and TaB$_{2}$ (Vegard’s law), which can be directly interpreted in terms of electronic band filling. Additionally, our prediction reveals that Sc$_{1-x}$Ta$_{x}$B$_{2}$ exhibits more superior mechanical properties than ScB$_{2}$ and TaB$_{2}$, and within a certain range of *x* it becomes superhard with hardness exceeding 40 GPa.

**Figure 1:**
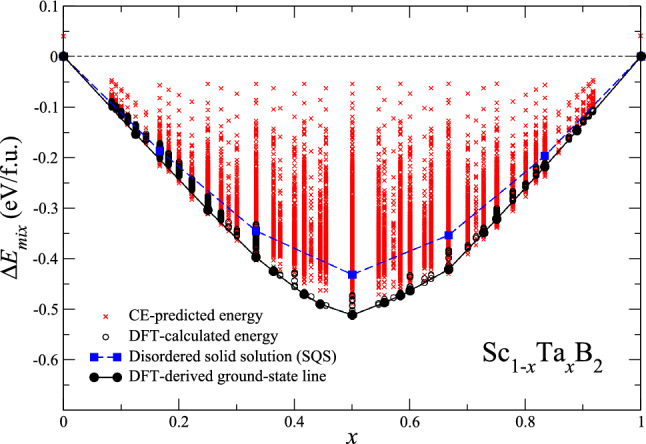
Energies of mixing ($\Delta$ $E_{mix}$) at *T* = 0 K of ordered and disordered solid solutions of Sc$_{1-x}$Ta$_{x}$B$_{2}$ evaluated with respect to ScB$_{2}$ and TaB$_{2}$. Red crosses denote the cluster-expansion (CE) predicted $\Delta$ $E_{mix}$ of 20240 ordered Sc$_{1-x}$Ta$_{x}$B$_{2}$ solid solutions. Open black circles represent the density-functional-theory (DFT) calculated $\Delta$ $E_{mix}$ of 1241 ordered Sc$_{1-x}$Ta$_{x}$B$_{2}$ solid solution, included in the final CE. Blue squares stand for the DFT-calculated $\Delta$ $E_{mix}$ of 5 disordered Sc$_{1-x}$Ta$_{x}$B$_{2}$ solid solutions, modeled by the SQS technique^29^. Thick black lines, connecting large filled black circles, indicate the ground-state lines of ordered Sc$_{1-x}$Ta$_{x}$B$_{2}$ solid solutions, derived from the DFT calculations.

## Results and discussion

### Mixing thermodynamics of Sc$_{1-x}$Ta$_{x}$B$_{2}$

We as a first step assess the alloying behavior of Sc and Ta atoms in the solid solutions of Sc$_{1-x}$Ta$_{x}$B$_{2}$. To this end, the cluster expansion of the total energy of Sc$_{1-x}$Ta$_{x}$B$_{2}$ according to the mathematical foundation of Sanchez, Ducastelle, and Grastias^30^ is performed, together with the Connolly-Williams method^31^, to determine the effective interactions between Sc and Ta. In the present work, our cluster-expansion model utilizes a total of 33 effective interactions (1 zerolet, 1 singlet, 19 pair, and 12 triplet interactions) and it fits the DFT-derived total energies of 1241 out of 20420 structures of ordered Sc$_{1-x}$Ta$_{x}$B$_{2}$, as the input of the model, with the leave-one-out cross validation score of 10.235 meV/f.u. The obtained effective interactions are then used for prediction of the total energies of the remaining 19179 structures of ordered Sc$_{1-x}$Ta$_{x}$B$_{2}$, not included in building the model. Figure 1 displays the energies of mixing ($\Delta$ $E_{mix}$) at *T* = 0 K of ordered and disordered solid solutions of Sc$_{1-x}$Ta$_{x}$B$_{2}$, calculated with respect to ScB$_{2}$ as well as TaB$_{2}$. The negative values of $\Delta$ $E_{mix}$ of Sc$_{1-x}$Ta$_{x}$B$_{2}$, where 0 $\leqslant$ *x* $\leqslant$ 1, indicate that ScB$_{2}$ and TaB$_{2}$ readily mix with each other, therefore resulting in the formation of Sc$_{1-x}$Ta$_{x}$B$_{2}$ solid solutions. As can be seen also from Fig. 1, $\Delta$ $E_{mix}$ of ScB$_{2}$, TaB$_{2}$, and ordered Sc$_{1-x}$Ta$_{x}$B$_{2}$ at different fixed values of *x* lie on the convex hull (a series of thick black lines connecting large filled blacked circles), suggesting that they are thermodynamically stable and promising candidates of ground-state structures for Sc$_{1-x}$Ta$_{x}$B$_{2}$. Also, we note here that the convex hull of the pseudo-binary ScB$_{2}$ $-$TaB$_{2}$ system, predicted by the cluster-expansion model, agrees with that derived from the DFT calculations. This, together with the relatively low cross-validation score of 10.235 meV/f.u., ensures the predictive ability of the model. Besides the ground-state structures of Sc$_{1-x}$Ta$_{x}$B$_{2}$, whose $\Delta$ $E_{mix}$ lie on the convex hull, we observe that $\Delta$ $E_{mix}$ of the lowest-energy structure of ordered Sc$_{1-x}$Ta$_{x}$B$_{2}$ of a given composition *x*, predicted to be unstable at *T* = 0 K and visualized in Fig. 1 as open black circles, lie only slightly above the convex hull by a few meV/f.u. Such tiny differences in $\Delta$ $E_{mix}$ between the lowest-energy structures of unstable-ordered Sc$_{1-x}$Ta$_{x}$B$_{2}$ and the convex hull are comparable to the numerical accuracy of our DFT total-energy calculations of Sc$_{1-x}$Ta$_{x}$B$_{2}$ and smaller than the cross-validation score of our cluster-expansion model. Our results and analyses on the mixing thermodynamics of ScB$_{2}$ and TaB$_{2}$ thus implies the thermodynamic stability of ordered Sc$_{1-x}$Ta$_{x}$B$_{2}$ over the entire composition range (0 $\leqslant$ *x* $\leqslant$ 1) even at *T* = 0 K. Our prediction of the mixing tendency of Sc and Ta atoms, residing on the metal sublattice of Sc$_{1-x}$Ta$_{x}$B$_{2}$, is qualitatively in line with the theoretical results, previously reported in the literature^2,27,28^. It is also worth mentioning that the formation of single-phase substitutional solid solutions of Sc$_{1-x}$Ta$_{x}$B$_{2}$ can be straightforwardly interpreted by the Hume-Rothery rules stating that the formation of solid solutions can be expected, if the atomic sizes and the electronegativity of the constituent elements differ by less than 15% and 0.4, respectively, and the crystal structures of the constituent elements must be similar^32^. For this particular case, the constituent elements of Sc$_{1-x}$Ta$_{x}$B$_{2}$ are ScB$_{2}$ and TaB$_{2}$, both of which are isostructural. Also, we find that the atomic radius of Ta is larger than that of Sc by about 8.7%, and the difference between their electronegativity is 0.14 only. This analysis, based on the Hume-Rothery rules, further strengthens the reliability of our results on the mixing thermodynamics of Sc and Ta atoms in Sc$_{1-x}$Ta$_{x}$B$_{2}$.

**Figure 2:**
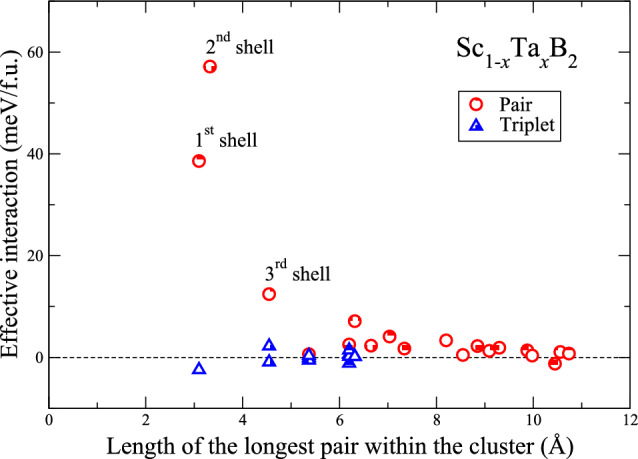
Effective pair and triplet interactions between transition-metal atoms residing on the metal sublattice of Sc$_{1-x}$Ta$_{x}$B$_{2}$ for several short-range coordination shells, derived from the final CE.

By inspecting the effective pair and triplet interactions between the metal atoms for several short-range coordination shells of the metal sublattice in Sc$_{1-x}$Ta$_{x}$B$_{2}$, as extracted from the cluster-expansion model and shown in Fig. 2, we find that the magnitudes of the pair interactions for the first, second, and third coordination shells are, respectively, 38.593, 57.154, and 12.442 meV/f.u. The strength of these pair interactions are relatively much stronger than those of the remaining pair and triplet interactions, whose magnitudes are lower than 7 meV/f.u. Because of the strong pair interactions between the metal atoms for the first three coordination shells in Sc$_{1-x}$Ta$_{x}$B$_{2}$, any Sc and Ta atoms constituting the solid solutions tend to be surrounded by metal atoms of the opposite type, residing in their first, second, and third coordination shells, in order to lower their total energies. That is, Sc$_{1-x}$Ta$_{x}$B$_{2}$ displays short-range chemical ordering of Sc and Ta atoms, leading to thermodynamic stabilization of ordered Sc$_{1-x}$Ta$_{x}$B$_{2}$, at low temperatures. It is worth noting that, as Sc$_{1-x}$Ta$_{x}$B$_{2}$ is subjected to high-temperature conditions, the ordered patterns of Sc and Ta atoms can become disordered due mainly to the increasingly strong contribution of mixing entropy ($\Delta$ $S_{mix}$), arising from random distribution of Sc and Ta atoms on the metal sublattice of Sc$_{1-x}$Ta$_{x}$B$_{2}$. Therefore, it is of interest to estimate the temperature, at which Sc and Ta atoms, residing on the metal sublattice of Sc$_{1-x}$Ta$_{x}$B$_{2}$, undergoes such an order-to-disorder transition across the whole composition range. In the present work, we do so by comparing the Gibbs free energies of mixing ($\Delta$ $G_{mix}$) of disordered solid solutions of Sc$_{1-x}$Ta$_{x}$B$_{2}$ to those of the ordered ones. Given that the effects of pressure, lattice vibrations, and electronic excitations are neglected, $\Delta$ $G_{mix}$ as a function of temperature *T* and chemical composition *x* can be expressed as;

1 $$\begin{aligned} \Delta G_{mix}(x,T) = \Delta E_{mix}(x) - T\Delta S_{mix}(x). \end{aligned}$$

To estimate $\Delta$ $G_{mix}$ for disordered Sc$_{1-x}$Ta$_{x}$B$_{2}$, the models of Sc$_{1-x}$Ta$_{x}$B$_{2}$ created by the SQS technique^29^ are employed, together with the DFT calculations, to obtain $\Delta$ $E_{mix}$ of the disordered solid solutions (blue shaded squares shown in Fig. 1), and their $\Delta$ $S_{mix}$ are analytically derived from the mean-field approach^12^. That is,

2 $$\begin{aligned} \Delta S_{mix}(x) = -k_B[x\ln (x)+(1-x)\ln (1-x)]. \end{aligned}$$

**Figure 3:**
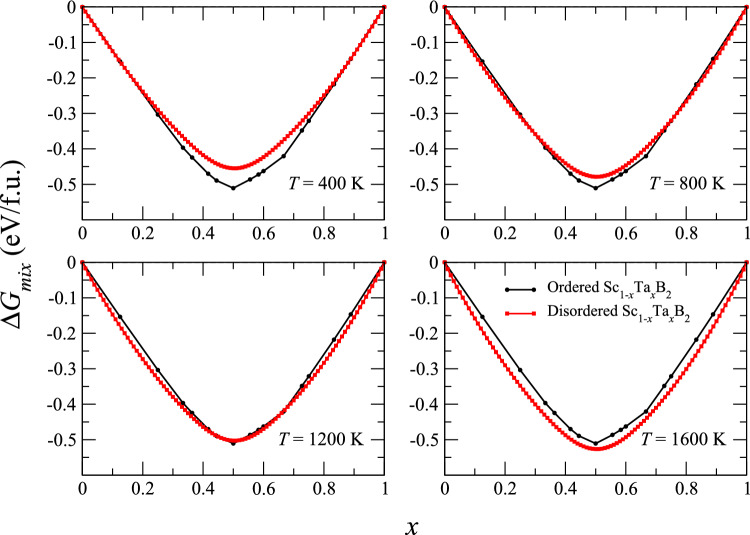
Gibbs free energies of mixing ($\Delta$ $G_{mix}$) of ground-state ordered (black filled circles) and disordered (red filled squares) structures of Sc$_{1-x}$Ta$_{x}$B$_{2}$ solid solutions, evaluated with respect to ScB$_{2}$ and TaB$_{2}$, at *T* = 400, 800, 1200, and 1600 K.

Note that, since our $\Delta$ $E_{mix}$ of disordered Sc$_{1-x}$Ta$_{x}$B$_{2}$ are calculated at 5 discrete compositions (*x* = 0.167, 0.333, 0.5, 0.667, and 0.833), they together with $\Delta$ $E_{mix}$ of ScB$_{2}$ (*x* = 0) and TaB$_{2}$ (*x* = 1) are interpolated by the cubic spline function and then combined with the term $-T$ $\Delta$ $S_{mix}$, where $\Delta$ $x$ = 0.01, to estimate $\Delta$ $G_{mix}$ for disordered Sc$_{1-x}$Ta$_{x}$B$_{2}$. Ordered solid solutions of Sc$_{1-x}$Ta$_{x}$B$_{2}$ are, on the other hand, assumed to exhibit the zero-Kelvin properties. Thus, $\Delta$ $S_{mix}$ for ordered Sc$_{1-x}$Ta$_{x}$B$_{2}$ are approximated to be zero, and their $\Delta$ $G_{mix}$ are provided solely by $\Delta$ $E_{mix}$ of the ground-state structures of Sc$_{1-x}$Ta$_{x}$B$_{2}$. That is, the DFT-derived ground-state lines of ordered Sc$_{1-x}$Ta$_{x}$B$_{2}$, shown in Fig. 1. Figure 3 illustrates the curves of $\Delta$ $G_{mix}$ for ground-state ordered and disordered structures of Sc$_{1-x}$Ta$_{x}$B$_{2}$ at four selected temperatures. We observe that $\Delta$ $G_{mix}$ curve of disordered Sc$_{1-x}$Ta$_{x}$B$_{2}$ lies below that of ground-state ordered Sc$_{1-x}$Ta$_{x}$B$_{2}$ across the entire composition range at *T* $\gtrsim$ 1337 K. This also implies that, above such a critical temperature, Sc and Ta atoms residing on the metal sublattice of Sc$_{1-x}$Ta$_{x}$B$_{2}$ configurationally disorder, and Sc$_{1-x}$Ta$_{x}$B$_{2}$ is thermodynamically stable in the form of single-phase disordered solid solutions for the whole composition range.

Note also that, because of the use of the mean-field approach to estimate $\Delta$ $S_{mix}$ for disordered Sc$_{1-x}$Ta$_{x}$B$_{2}$, modeled by the SQS method, the configurational order-to-disorder transition temperature of Sc$_{1-x}$Ta$_{x}$B$_{2}$ can be overestimated by 20–30%, approximately^33–35^. This can be explained by the absence of the short-range ordering of Sc and Ta atoms in the SQS models of Sc$_{1-x}$Ta$_{x}$B$_{2}$ and the error in the mean-field estimated $\Delta$ $S_{mix}$. The other sources of uncertainty in prediction of the order-to-disorder transition temperature may be attributed to the neglect of effects, arising for example from lattice vibrations and electronic excitations. It has been shown that, without explicitly taking into account the effect of lattice vibrations, the configurational order-to-disorder transition temperature of a given alloy system can be overestimated by up to 30%^36,37^. Based on this error analysis, one can reasonably expect that the order-to-disorder transition temperature of Sc$_{1-x}$Ta$_{x}$B$_{2}$, derived here from the mean-field approach, is overestimated by a few hundred Kelvin. Despite ordered Sc$_{1-x}$Ta$_{x}$B$_{2}$, predicted to be thermodynamically stable at *T* $\lesssim$ 1337 K, disordered solid solutions of Sc$_{1-x}$Ta$_{x}$B$_{2}$ may be experimentally achieved in the form of thin solid films at moderate-to-low temperatures by using, for example, magnetron sputtering techniques enabling kinetic limitation^4,6–8,10,11,13^, and thus the as-fabricated films of disordered Sc$_{1-x}$Ta$_{x}$B$_{2}$ could remain metastable under ambient conditions.

**Figure 4:**
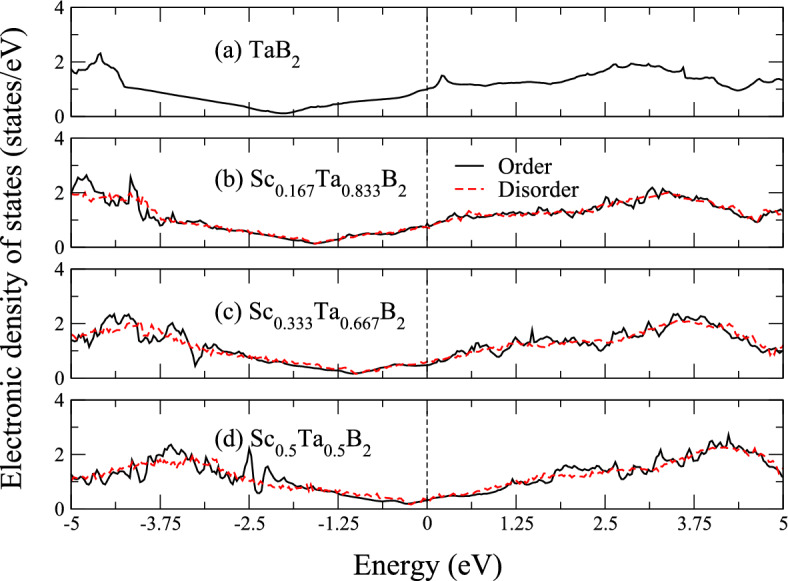
Electronic density of states of ordered (black solid line) and disordered (red dashed line) Sc$_{1-x}$Ta$_{x}$B$_{2}$ with *x* = 0.5, 0.667, 0.833, and 1. The vertical dashed line at 0 eV in (a)−(d) indicates the highest occupied electronic state.

**Figure 5:**
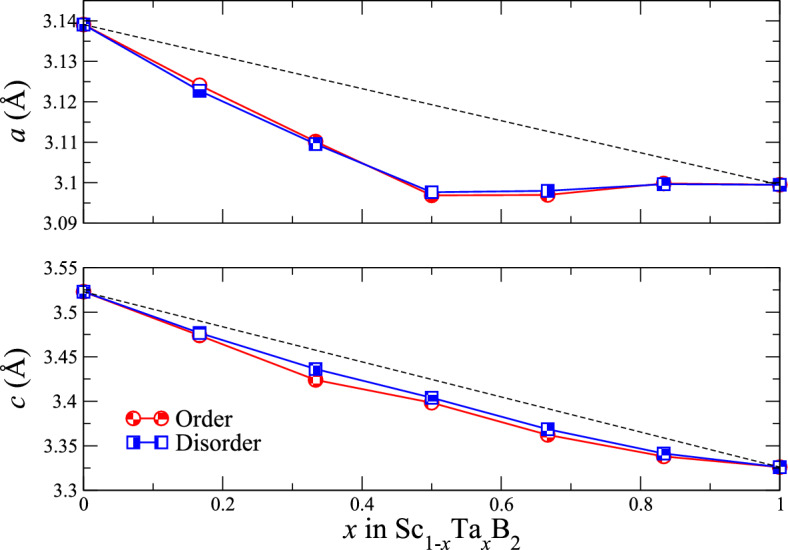
Equilibrium lattice parameters (*a* and *c*) of ordered (red shaded circles) and disordered (blue shaded squares) solid solutions of Sc$_{1-x}$Ta$_{x}$B$_{2}$, where 0 $\leqslant$ *x* $\leqslant$ 1 and $\Delta$ $x$ = 1/3. The black dashed lines are evaluated between ScB$_{2}$ (*x* = 0) and TaB$_{2}$ (*x* = 1) indicate the linear Vegard’s law.

### Electronic, structural and mechanical properties of Sc$_{1-x}$Ta$_{x}$B$_{2}$

As demonstrated and discussed in the theoretical works on metal diborides, previously published in the literature^12,14–16,18,19,38^, the negative values of $\Delta$ $G_{mix}$ for both ordered and disordered Sc$_{1-x}$Ta$_{x}$B$_{2}$ solid solutions, indicating their thermodynamic stability relative to ScB$_{2}$ and TaB$_{2}$, can be directly explained by the changes in the number of electrons filling bonding and antibonding states of Sc$_{1-x}$Ta$_{x}$B$_{2}$. Due to the interactions between metal atoms arranging themselves in the simple hexagonal geometry^2^, as in the case of metal diborides, the electronic density of states of the diborides displays a valley-like feature, frequently separating the bonding states from the antibonding states, around the Fermi level^14,15,18,38–40^. For ScB$_{2}$, the valley is located above the Fermi level, implying that there exist still unfilled bonding states of the material^12,15,18,39^. This can also be confirmed by considering the corresponding chemical bondings of the compound, visualized through its crystal orbital Hamilton population (−COHP)^18,41^. As can be seen from Fig. S1, the −COHP bonding analysis for ScB$_{2}$ clearly show non-zero bonding states at the Fermi level. On the other hand, the electronic density of states of TaB$_{2}$ apparently reveal that the valley lies below the Fermi level^15,18,19,39,42^. This indicates that, for TaB$_{2}$, not only the bonding states of the material are fully occupied, but some of its antibonding states resulting particularly from the interactions between the 5*d* orbitals of Ta atoms and the 2*p* orbitals of B atoms are also filled by electrons^39,43^, see also Fig. S2 illustrating the −COHP bonding analysis for TaB$_{2}$. Figure 4 shows the electronic density of states around the Fermi level of Sc$_{1-x}$Ta$_{x}$B$_{2}$ with *x* = 0.5, 0.667, 0.833, and 1. We find that partial substitution of Sc atoms for Ta atoms in TaB$_{2}$, leading to the formation of Sc$_{1-x}$Ta$_{x}$B$_{2}$ solid solutions, can reduce the number of electrons occupying the diboride’s antibonding states. As the value of *x* in Sc$_{1-x}$Ta$_{x}$B$_{2}$ decreases from 1 to 0.5, the Fermi level shifts toward the valley’s bottom and the number of electronic states at the Fermi level decreases as compared to that of TaB$_{2}$. Such a shift of the Fermi level toward to the valley due to the partial replacement of some Ta atoms in TaB$_{2}$ by Sc atoms is an indication of a decrease in the number of electron filling the material’s antibonding states, and it is verified, for example, by the −COHP bonding analysis for the ordered solid solution of Sc$_{0.5}$Ta$_{0.5}$B$_{2}$, as shown in Fig. S3. It is worth noting here that our results on the −COHP bonding analysis for ScB$_{2}$ and TaB$_{2}$ are in good agreement with those, recently reported by Dahlqvist et al.^18^. The depletion of electrons in the antibonding states is in turn expected to result in the strengthening of bonding between the atoms constituting Sc$_{1-x}$Ta$_{x}$B$_{2}$. The shift of the Fermi level toward the valley and the reduction of the number of electronic states at the Fermi level can also be observed for ScB$_{2}$, when some Sc atoms residing on the metal sublattice of the compound are replaced by Ta atoms (*not* *shown*). Nevertheless, we note that the changes in the electronic properties in this case is related to an increase in the number of electrons filling the bonding states of the material. We note further that the rigid-band model can herein be assumed when describing any change in the electronic properties of Sc$_{1-x}$Ta$_{x}$B$_{2}$, induced by variation of ScB$_{2}$ and TaB$_{2}$ contents. Besides, we observe that, for a given value of *x*, the electronic density of states around the Fermi level of disordered Sc$_{1-x}$Ta$_{x}$B$_{2}$ imitates that of ordered Sc$_{1-x}$Ta$_{x}$B$_{2}$ (see Fig. 4), suggesting that the configuration of Sc and Ta atoms residing on the metal sublattice has minimal impact on the electronic properties of Sc$_{1-x}$Ta$_{x}$B$_{2}$. Based on these findings, we propose that such an effect of band filling, induced by alloying TaB$_{2}$ with Sc and *vice* *versa*, is essentially responsible for the thermodynamic stability of Sc$_{1-x}$Ta$_{x}$B$_{2}$ solid solutions with respect to their constituent compounds. According to our prediction, the stability of Sc$_{1-x}$Ta$_{x}$B$_{2}$ is expected to be at maximum at *x* $\thickapprox$ 0.5. This is because, at such a composition, its Fermi level is positioned very close to the valley’s bottom and the number of electronic states at the Fermi level is minimum.

The band filling-induced improvement in the bond strength between the atoms constituting Sc$_{1-x}$Ta$_{x}$B$_{2}$ may be characterized by negative deviation of their lattice parameters *a* and *c* from the linear mixing trends, drawn between the parameters *a* and *c* of ScB$_{2}$ and TaB$_{2}$. As can be seen from Fig. 5, the parameters *a* and *c* of (either ordered or disordered) Sc$_{1-x}$Ta$_{x}$B$_{2}$ are negatively deviating from the linear Vegard’s rule by up to 0.7% and 0.9%, respectively. On the other hand, the parameters *a* and *c* of disordered Sc$_{1-x}$Ta$_{x}$B$_{2}$ differ from those of ordered Sc$_{1-x}$Ta$_{x}$B$_{2}$ by less than 0.05% and 0.4%, respectively. To the best of our knowledge, we are not aware of any experimental and theoretical works reporting the values of *a* and *c* for Sc$_{1-x}$Ta$_{x}$B$_{2}$, except for ScB$_{2}$ and TaB$_{2}$. We note that the reliability of our approach used to derive the properties of ScB$_{2}$ and TaB$_{2}$, including the lattice parameters, has already been demonstrated and discussed in our theoretical works on Sc$_{1-x}$V$_{x}$B$_{2}$^12^ and B-deficient TaB$_{2-x}$^19^, in which our calculated values of lattice parameters (both *a* and *c*) of the two diborides were compared and found to be in excellent agreement with the existing experimental and theoretical data, available in the literature^38,43–48^.

**Figure 6:**
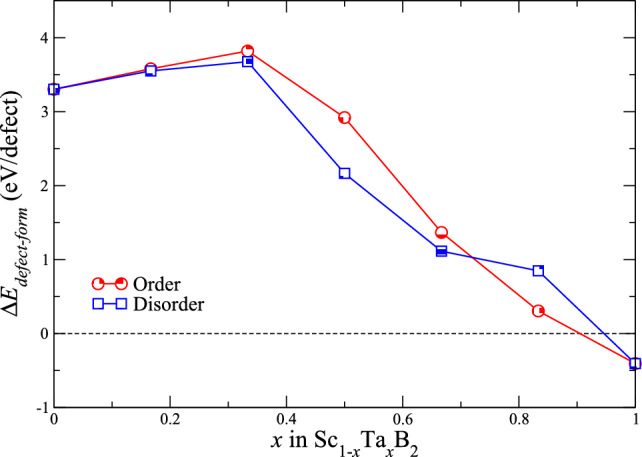
Formation energies ($\Delta$ $E_{defect-form}$) of B vacancy in the dilute limit of ordered (red shaded circles) and disordered (blue shaded squares) solid solutions of Sc$_{1-x}$Ta$_{x}$B$_{2}$, where 0 $\leqslant$ *x* $\leqslant$ 1 and $\Delta$ $x$ = 1/3.

The effect of electronic band filling has lately been theoretically shown to play an important role also in determining the mechanical properties of metal diborides^12,15,16,19^. In case of double-metal diborides, like Sc$_{1-x}$Ta$_{x}$B$_{2}$, the effect can be triggered for example by controlling the contents of ScB$_{2}$ and TaB$_{2}$ and/or by the presence of structural defects. Recent theoretical works on metal diborides^18,19^ revealed that, for TaB$_{2}$ crystallizing in the AlB$_{2}$-type structure, the effect of band filling can be triggered by partial substitution of vacancies for B atoms. The presence of sufficiently small amount of such vacancies in TaB$_{2}$ results in the depletion of electrons in the antibonding states of the compound, and thus the thermodynamic stabilization of B-deficient TaB$_{2-x}$, where 0.167 $\lesssim$ *x* $\lesssim$ 0.25, even at absolute zero with superior mechanical properties as compared to those of stoichiometric TaB$_{2}$^19^. For this reason, we are motivated to examine the formation of B vacancy (in the dilute limit) in the solid solutions of Sc$_{1-x}$Ta$_{x}$B$_{2}$, as characterized by the defect formation energy ($\Delta$ $E_{defect-form}$). For a given configuration of Sc$_{1-x}$Ta$_{x}$B$_{2}$, $\Delta$ $E_{defect-form}$ can be evaluate from;

3 $$\begin{aligned} \Delta E_{defect-form}=E_{defect} - (E_{defect-free} - \mu _{B}), \end{aligned}$$

where $E_{defect-free}$ and $E_{defect}$ denote the total energies of defect-free and defective Sc$_{1-x}$Ta$_{x}$B$_{2}$, respectively, and $\mu _{B}$ is the chemical potential of B, which is derived from the total energy per atom of $\alpha$-rhombohedral B. Herein, the structural model of any defect-free Sc$_{1-x}$Ta$_{x}$B$_{2}$ is modeled in within a 144-atom supercell, and the corresponding defective structure is created by removing a single B atom from the defect-free supercell of Sc$_{1-x}$Ta$_{x}$B$_{2}$. As can be seen from Fig. 6, showing $\Delta$ $E_{defect-form}$ of B vacancy in the dilute limit of ordered and disordered Sc$_{1-x}$Ta$_{x}$B$_{2}$ with *x* ranging from 0 to 1, $\Delta$ $E_{defect-form}$ of B vacancy is about −0.4 eV/defect for TaB$_{2}$. The negative sign for $\Delta$ $E_{defect-form}$ of TaB$_{2}$ indicates that, for this particular compound, a small amount of B vacancies is thermodynamically favored, and some B atoms residing on the boron sublattice of the diboride are thus readily be substituted by vacancies. Nevertheless, as one-sixth of Ta atoms residing on the metal sublattice of the diboride are replaced by Sc atoms, giving rise to the formation of either ordered or disordered solid solution of Sc$_{0.167}$Ta$_{0.833}$B$_{2}$, $\Delta$ $E_{defect-form}$ of B vacancy becomes positive, and its value considerably increases up to about +3.8 eV/defect with *x* decreasing from 0.833 to 0.333, before it slightly decreases to a value of +3.3 eV/defect at *x* equal to 0. The change from the negative sign to the positive one of $\Delta$ $E_{defect-form}$ of B vacancy in the dilute limit of the diboride, as Ta atoms are (partially or fully) substituted by Sc atoms, indicates that introducing Sc atoms into TaB$_{2}$ can hinder the formation of B vacancies in the pseudo-binary alloy system of Sc$_{1-x}$Ta$_{x}$B$_{2}$. Accordingly, the concentration of B vacancies in Sc$_{1-x}$Ta$_{x}$B$_{2}$ with $x < 1$ is expected to be very tiny, as compared to that in TaB$_{2}$. By considering this, together with the positive and high values of $\Delta$ $E_{defect-form}$ for other types of structural defects in ScB$_{2}$ and TaB$_{2}$ in the dilute limit as reported in the previous studies of Dahlqvist et al.^18^ and Ektarawong et al.^19^, it is reasonable for us to study the mechanical properties of Sc$_{1-x}$Ta$_{x}$B$_{2}$ solid solutions at different fixed values of *x* without taking into account any structural defects in the models of ordered and disordered Sc$_{1-x}$Ta$_{x}$B$_{2}$.

**Figure 7:**
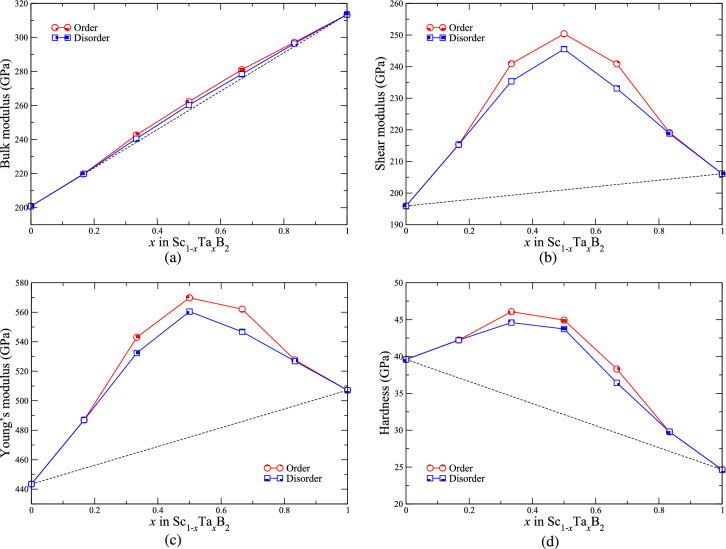
(**a**) Bulk, (**b**) shear, and (**c**) Young’s moduli, as well as (**d**) hardness of ordered (red shaded circles) and disordered (blue shaded squares) solid solutions of Sc$_{1-x}$Ta$_{x}$B$_{2}$ with *x* = 0, 0.167, 0.333, 0.5, 0.667, 0.833, and 1. The black dashed lines in (**a**)–(**d**) are drawn by assuming the linear Vegard’s law between ScB$_{2}$ (*x* = 0) and TaB$_{2}$ (*x* = 1).

**Figure 8:**
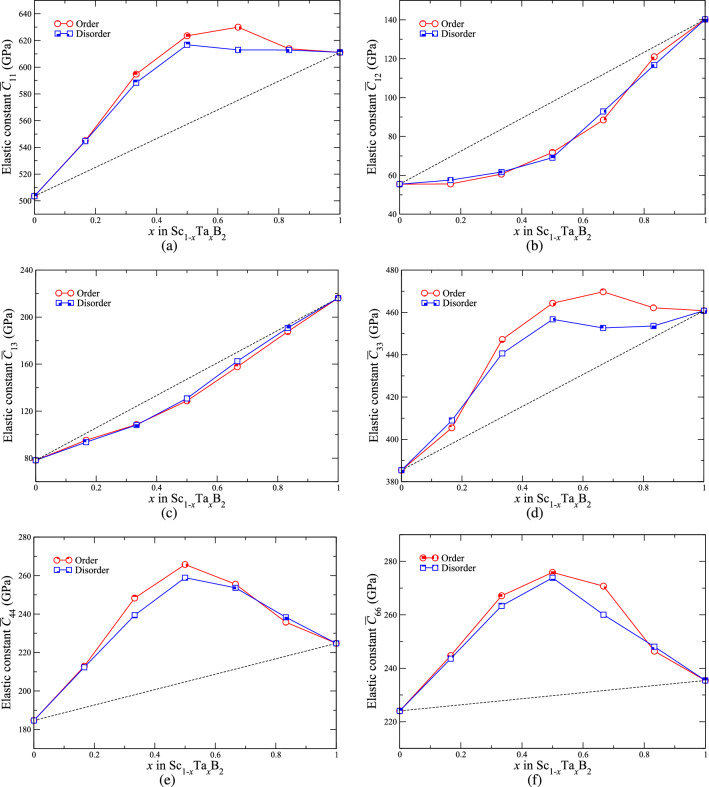
Projected hexagonal elastic constants $\bar{C}_{ij}$ of ordered (red shaded circles) and disordered (blue shaded squares) solid solutions of Sc$_{1-x}$Ta$_{x}$B$_{2}$, where 0 $\leqslant$ *x* $\leqslant$ 1 and $\Delta$ $x$ = 1/3. The black dashed lines in (**a**)–(**f**) are drawn by assuming the linear Vegard’s law between ScB$_{2}$ (*x* = 0) and TaB$_{2}$ (*x* = 1).

Figures 7 and 8 illustrate the values of the elastic moduli and constants as well as the hardness of ordered and disordered Sc$_{1-x}$Ta$_{x}$B$_{2}$ with *x* = 0, 0.167, 0.333, 0.5, 0.667, 0.833, and 1. Just as in the case of lattice parameters *a* and *c*, we are not aware of any experimental and theoretical works on metal diborides reporting the values of the elastic moduli and constants as well as the hardness for Sc$_{1-x}$Ta$_{x}$B$_{2}$, where 0 $< x<$ 1. We have therefore to verify the reliability of our theoretical results on the mechanical properties of Sc$_{1-x}$Ta$_{x}$B$_{2}$ solid solutions by making a comparison only with the experimentally measured and theoretically calculated mechanical properties of ScB$_{2}$ and TaB$_{2}$, found in the literature. We have shown in our previous works on Sc$_{1-x}$V$_{x}$B$_{2}$^12^ and boron-deficient TaB$_{2-x}$^19^ that the mechanical properties of metal diborides, including ScB$_{2}$ and TaB$_{2}$, derived from our techniques, are quantitatively in line with the experimental and theoretical data on the mechanical properties of the two diborides, as previously reported in the literature^43,48–54^. Evidently, this not only validates our results of the mechanical properties of ScB$_{2}$ and TaB$_{2}$, but it also gives us the confidence of implementing such a methodology to evaluate the mechanical properties of Sc$_{1-x}$Ta$_{x}$B$_{2}$ solid solutions. As can be seen from Fig. 7, the three elastic moduli as well as the hardness Sc$_{1-x}$Ta$_{x}$B$_{2}$ solid solutions reveal positive deviation from the linear Vegard’s law evaluated between those of ScB$_{2}$ and TaB$_{2}$. This indicates the enhancement of mechanical properties of Sc$_{1-x}$Ta$_{x}$B$_{2}$ solid solutions with respect to their constituent compounds. Even though the values of bulk modulus of Sc$_{1-x}$Ta$_{x}$B$_{2}$ are found to slightly deviate from Vegard’s law by no larger than 2%, the shear and Young’s moduli as well as the hardness of the solid solutions significantly and largely deviate from the linear mixing rule. Interestingly, the degrees of deviation can be as high as 25%, 20%, and 40% for shear modulus, Young’s modulus, and hardness, respectively, and for the alloy system of Sc$_{1-x}$Ta$_{x}$B$_{2}$ the degrees of deviation are highest at *x* = 0.5. We also observe that the hardness of Sc$_{1-x}$Ta$_{x}$B$_{2}$, where 0.167 $\lesssim$ *x* $\lesssim$ 0.5, is higher than 40 GPa, indicating the superhard properties of Sc$_{1-x}$Ta$_{x}$B$_{2}$ within such a range of composition. The changes in the positive direction both of the elastic moduli and of the hardness of Sc$_{1-x}$Ta$_{x}$B$_{2}$ with respect to the linear mixing trends drawn between those of ScB$_{2}$ and TaB$_{2}$ are related to the significant deviation from the Vegard’s rule of the elastic constants $\bar{C}_{ij}$, in particular $\bar{C}_{12}$ and $\bar{C}_{44}$, whose degrees of deviation can be as large as 29% and 26%, respectively, as shown in Fig. 8. The significant improvement in the mechanical properties, especially the shear and Young’s moduli as well as the hardness, of Sc$_{1-x}$Ta$_{x}$B$_{2}$ with respect to ScB$_{2}$ and TaB$_{2}$ is indeed a directly consequence of the effect of electronic band filling, induced upon mixing TaB$_{2}$ with ScB$_{2}$, and it corresponds to the negative deviation of the parameters *a* and *c* of Sc$_{1-x}$Ta$_{x}$B$_{2}$ from their linear mixing trends (see also Fig. 5), implying the increase in the bond strength between the atoms constituting Sc$_{1-x}$Ta$_{x}$B$_{2}$. We additionally observed the differences in the values of elastic constants, elastic moduli, and hardness between ordered and disordered Sc$_{1-x}$Ta$_{x}$B$_{2}$, for a given value of *x*, are not exceeding 5%. The similarity between the electronic, structural, and mechanical properties of ordered and disordered Sc$_{1-x}$Ta$_{x}$B$_{2}$, both having the same composition, does suggest that the arrangement of Sc and Ta atoms in the metal sublattice of Sc$_{1-x}$Ta$_{x}$B$_{2}$ probably has a small impact on its physical properties, similar to the cases of Sc$_{1-x}$V$_{x}$B$_{2}$^12^ and boron-deficient TaB$_{2-x}$^19^. It should further be mentioned that, for ordered and disordered Sc$_{1-x}$Ta$_{x}$B$_{2}$, the Born stability criteria for any materials crystallizing in the hexagonal structure^55^ are fulfilled for all values of *x*, suggesting that they are mechanically stable. On the other hand, the bulk-to-shear moduli ratio of Sc$_{1-x}$Ta$_{x}$B$_{2}$ is found to range, approximately, from 1 to 1.5 depending on the value of *x*. Based on the Pugh’s criteria^56^ widely used to distinguish the ductility and brittleness of materials in terms of the bulk-to-shear moduli ratio, Sc$_{1-x}$Ta$_{x}$B$_{2}$ should behave as an intrinsically brittle material. The significant enhancement of the mechanical properties and the stabilities of Sc$_{1-x}$Ta$_{x}$B$_{2}$ with respect to ScB$_{2}$ and TaB$_{2}$ further highlights the important role of band filling, induced by selective mixing of two (or more than two) distinct metal diborides, in designing stable double-metal (or multi-metal) diborides with superior and tunable properties for hard-coating applications.

## Conclusion

In summary, we perform first-principles calculations, based particularly on the density functional theory and the cluster-expansion method, to elucidate the mixing thermodynamics of ScB$_{2}$ and TaB$_{2}$. Our findings reveal that ScB$_{2}$ and TaB$_{2}$ readily mix with each other, thus resulting in the formation of a single-phase solid solution of Sc$_{1-x}$Ta$_{x}$B$_{2}$ over the entire range of composition even at *T* = 0 K. The thermodynamic stability of Sc$_{1-x}$Ta$_{x}$B$_{2}$ relative to its constituent compounds can be attributed to the effect of electronic band filling, induced upon mixing TaB$_{2}$ with ScB$_{2}$, and the formation of the solid solutions of Sc$_{1-x}$Ta$_{x}$B$_{2}$ is found to be in accordance with the Hume-Rothery rules. We further find that such a band filling scenario controlled by variation of ScB$_{2}$ and TaB$_{2}$ contents plays a significant role in determining and to a large extent improving the mechanical properties, particularly the shear and Young’s moduli as well as the hardness, of Sc$_{1-x}$Ta$_{x}$B$_{2}$ solid solutions. By alloying TaB$_{2}$ with ScB$_{2}$, the values of the elastic moduli and the hardness of Sc$_{1-x}$Ta$_{x}$B$_{2}$ largely deviate in the positive direction from the linear mixing trends, evaluated by assuming the Vegard’s rule between ScB$_{2}$ and TaB$_{2}$. In case of Sc$_{1-x}$Ta$_{x}$B$_{2}$, the degrees of deviation from such linear trends can be up to 25, 20, and 40% for shear modulus, Young’s modulus, and hardness, respectively. These results not only highlight the importance of band-filling effects, induced by selective mixing of two (or more than two) distinct metal diborides, on the stability and the properties of double-metal (or multi-metal) diborides, but it can also offer a path to efficiently design thin films of metal diboride-based solid solutions with superior and widely tunable mechanical properties, suitable for relevant applications.

## Methods

A set of structural models of ordered solid solutions of Sc$_{1-x}$Ta$_{x}$B$_{2}$ with 0 $\leqslant$ *x* $\leqslant$ 1 are generated *via* the algorithm of Hard and Forcade^57^ as implemented in the MIT *Ab* *initio* Phase Stability (MAPS) code^58^ packaged with the Alloy-Theoretical Automated Toolkit (ATAT)^59^. In the present work, apart from ScB$_{2}$ and TaB$_{2}$, each modeled within the 3-atom primitive hexagonal unit cells, various shapes and sizes (with up to 36 atoms) primitive supercells are used for modeling the ordered solid solutions of Sc$_{1-x}$Ta$_{x}$B$_{2}$. This results totally in 20420 unique models of ordered Sc$_{1-x}$Ta$_{x}$B$_{2}$. Additionally, the special quasirandom structure (SQS) technique^29^ is employed to construct 144-atom hexagonal supercells of disordered solid solutions of Sc$_{1-x}$Ta$_{x}$B$_{2}$ with *x* = 0.167, 0.333, 0.5, 0.667, and 0.833. The projector augmented-wave method^60^ with the plane-wave energy cut-off of 500 eV, as implemented in the Vienna *Ab* *initio* Simulation Package (VASP)^61,62^, and the generalized gradient approximation, as proposed by Perdew, Burke, and Ernzherhof^63^, are employed to perform the total-energy calculations for Sc$_{1-x}$Ta$_{x}$B$_{2}$ based on the density functional theory (DFT)^64,65^. For each considered model of Sc$_{1-x}$Ta$_{x}$B$_{2}$, the volume and shape of its unit cell (or supercell) as well as the coordinates of all atoms in the cell are optimally relaxed during the DFT calculations to obtain its total energy in equilibrium, which is made sure to numerically converged within 1 meV/atom with respect to the value of the plane-wave energy cut-off and the number of Monkhorst−Pack ${\textbf {k}}$-point grids for sampling the Brillouin zone^66^.

The cluster-expansion formalism^30^ and the Connolly-Williams method^31^ as executed also in the MAPS code are employed to identify, among the generated 20420 structures of ordered Sc$_{1-x}$Ta$_{x}$B$_{2}$, candidates for ground-state structures of the solid solutions. Since the solid solutions are formed merely through the mixing of Sc and Ta atoms on the metal sublattice, B atoms are all taken as spectators and not thus included in any procedure for the determination of the ground-state structures of Sc$_{1-x}$Ta$_{x}$B$_{2}$. To determine the projected hexagonal elastic constants $\bar{C_{ij}}$ (in Voigt’s notation) for Sc$_{1-x}$Ta$_{x}$B$_{2}$ of a given structure, strains $\epsilon _{i}$ with ±1% and ±2% distortions are as a first step applied to its unit cell (or supercell) in equilibrium without volume conservation, and the constants $\bar{C_{ij}}$ are then derived from the second derivative of the total energy of Sc$_{1-x}$Ta$_{x}$B$_{2}$ under consideration as expressed in terms of power series of the strain vector ${\varvec{\varepsilon }}$ = {$\varepsilon _{1}$, $\varepsilon _{2}$, $\varepsilon _{3}$, $\varepsilon _{4}$, $\varepsilon _{5}$  $\varepsilon _{6}$} with respect to strains $\varepsilon _{i}$^67^, together with the implementation of the symmetry-based projection technique^68^. Note that, in the present work, the total energies of Sc$_{1-x}$Ta$_{x}$B$_{2}$ of a given structure evaluated at different degrees of distortion for a given applied strains $\epsilon _{i}$ are obtained from the DFT calculations, and fitted to the quadratic function. For a given structure of Sc$_{1-x}$Ta$_{x}$B$_{2}$, the obtained $\bar{C_{ij}}$ are in turn used to determine the polycrystalline bulk, shear, and Young’s moduli of *via* the Voigt-Reuss-Hill method^69^, while the hardness is derived from the bulk and shear moduli by using the model suggested by Chen et al.^70^. To derive the electronic density of states of Sc$_{1-x}$Ta$_{x}$B$_{2}$, the tetrahedron method with Blöchl corrections^71^ is chosen for the numerical integration of the Brillouin zone during the DFT calculations. As a complement, the chemical bondings between pairs of Sc, Ta and B atoms, constituting Sc$_{1-x}$Ta$_{x}$B$_{2}$, are described *via* the crystal orbital Hamilton population (−COHP), retrieved from the LOBSTER code^72–75^. Positive and negative values of −COHP indicate bonding and antibonding interactions, respectively.

## Supplementary Information

Supplementary Information.

## Data Availability

The data that support the findings of this study are available from the corresponding author upon reasonable request.
