# Supplementary material for: First-principles demonstration of band filling-induced significant improvement in thermodynamic stability and mechanical properties of Sc1-xTaxB2 solid solutions
Source: Sci Rep. 2023 Jun 28;13:10504. doi: 10.1038/s41598-023-37642-8 (PMC10307902; doi:10.1038/s41598-023-37642-8)
Supplement: Supplementary file 1 — Supplementary Information. [file 41598_2023_37642_MOESM1_ESM.pdf]

Supplementary Information

First-principles demonstration of band filling-induced significant improvement in thermodynamic stability and mechanical properties of $Sc_{1-x}Ta_xB_2$ solid solutions

Kunpot Mopoung[1], Annop Ektarawong[1,2,*], Thiti Bovornratanaraks[1], and Björn Alling[3]

[1] Extreme Condition Physics Research Laboratory and Center of Excellence in Physics of Energy Materials, Department of Physics, Faculty of Science, Chulalongkorn University, Bangkok, 10330, Thailand

[2] Chula Intelligent and Complex System, Faculty of Science, Chulalongkorn University, Bangkok, 10330, Thailand

[3] Theoretical Physics Division, Department of Physics, Chemistry and Biology (IFM), Linköping University, SE-581 83 Linköping, Sweden

* Corresponding author E-mail: Annop.E@chula.ac.th

1. Supplementary Figures

Figure S1

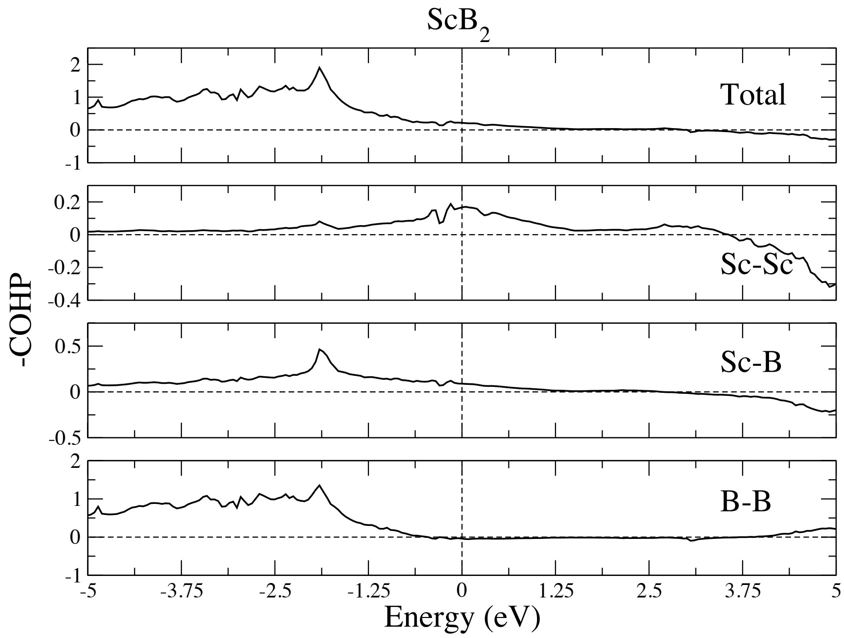

Figure S1. -COHP bonding analysis of Sc-Sc, Sc-B, B-B, and total for ScB_2. Positive and negative values of -COHP indicate, respectively, bonding and antibonding interactions. The vertical dashed lines at 0 eV indicate the highest occupied electronic states.

Figure S2

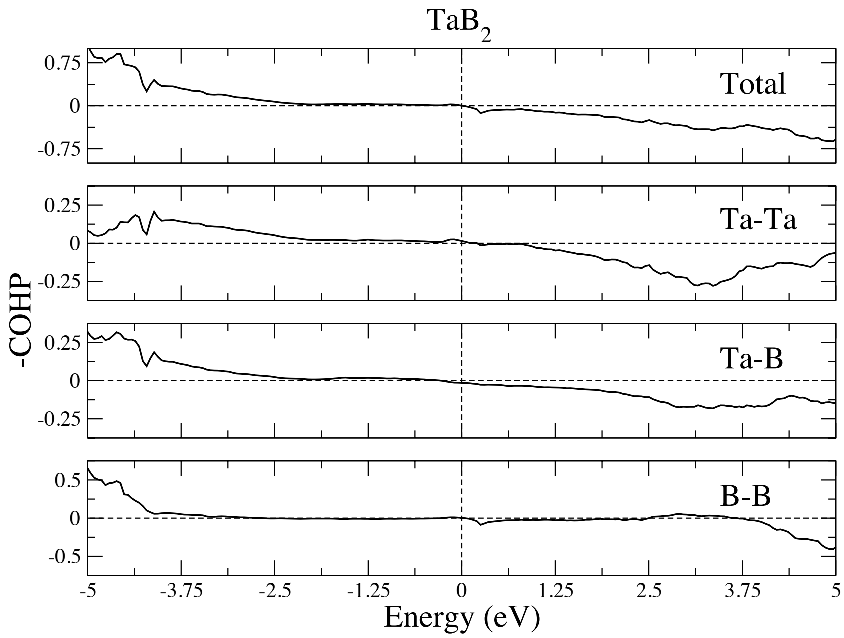

Figure S2. -COHP bonding analysis of Ta-Ta, Ta-B, B-B, and total for TaB_2. Positive and negative values of -COHP indicate, respectively, bonding and antibonding interactions. The vertical dashed lines at 0 eV indicate the highest occupied electronic states.

Figure S3

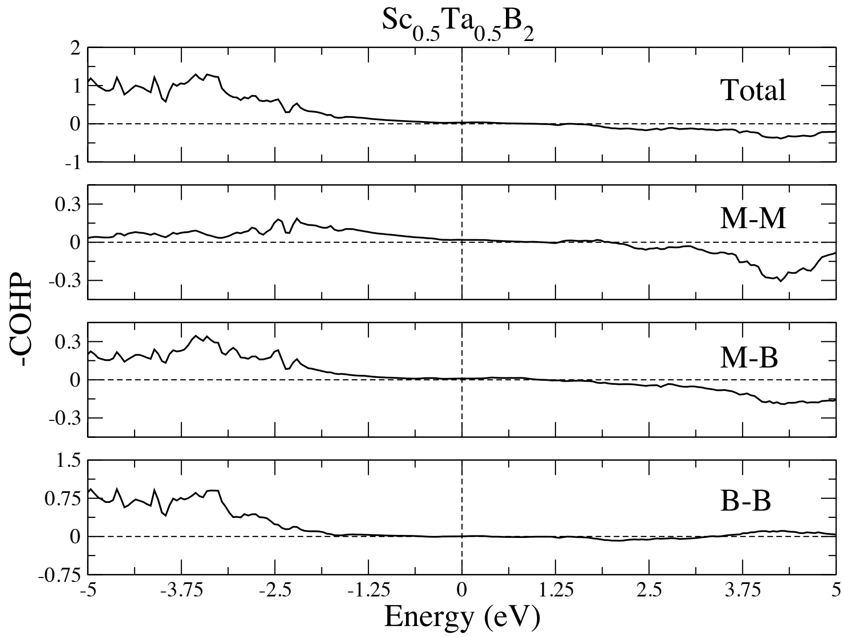

Figure S3. On average -COHP bonding analysis of M-M, M-B, B-B, and total for ordered $Sc_{0.5}Ta_{0.5}B_2$, where M = Sc and Ta. Positive and negative values of -COHP indicate, respectively, bonding and antibonding interactions. The vertical dashed lines at 0 eV indicate the highest occupied electronic states.
